# Unraveling the impacts of meteorological and anthropogenic changes on sediment fluxes along an estuary-sea continuum

**DOI:** 10.1038/s41598-021-99502-7

**Published:** 2021-10-12

**Authors:** Florent Grasso, Eliott Bismuth, Romaric Verney

**Affiliations:** Ifremer – DYNECO/DHYSED, Centre de Bretagne, CS 10070, 29280 Plouzané, France

**Keywords:** Physical oceanography, Sedimentology

## Abstract

Sediment fluxes at the estuary-sea interface strongly impact particle matter exchanges between marine and continental sources along the land-sea continuum. However, human activities drive pressures on estuary physical functioning, hence threatening estuarine habitats and their ecosystem services. This study explores a 22-year numerical hindcast of the macrotidal Seine Estuary (France), experiencing contrasted meteorological conditions and anthropogenic changes (i.e., estuary deepening and narrowing). The hindcast was thoroughly validated for both water column and sediment bed dynamics and showed good capacities to simulate annual sediment budgets observed from 1990 to 2015. We aim at disentangling the relative contributions of meteorological and human-induced morphological changes on net sediment fluxes between the estuary and its adjacent coastal sea. Our results highlight that intense wave events induce fine sediment (≤ 100 µm) export to the sea but coarser sediment (≥ 210 µm) import within the estuary. Although intense river discharges induce mud export to the sea, moderate to large river discharges prove to support mud import within the estuary. Wave and river discharge events were less intense in 2005–2015 than in 1990–2000, reducing fine sediment export to the sea. The estuary deepening and narrowing due to human activities increased fine sediment import within the estuary, shifting the estuary from an exporting to importing system. We propose a conceptualization of mud flux response to river discharge and wave forcing, as well as anthropogenic pressures. It provides valuable insights into particle transfers along the land-sea continuum, contributing to a better understanding of estuarine ecosystem trajectories under global changes.

## Introduction

Suspended sediments are vectors of nutrients and pollutants along the land-sea continuum^1^. In tidal estuaries, at the interface between continental freshwaters and coastal seas, sediment from diverse sources (e.g., marine, estuarine, and riverine supplies) are transported by river and tidal flows. The interaction of tide-induced and density-induced processes can trap sediment in estuary channels and intertidal flats, and form estuarine turbidity maxima (ETM)^2–8^. Such pools of mainly muddy sediment buffer particulate and dissolved matter exchange between terrigenous and marine sources, altering the system morphology and its functioning^9^. These dynamics directly impact estuarine habitats, which are considered among the most productive in the world^10,11^. In addition, estuaries often represent highly-populated areas and are strongly vulnerable to human pressures (e.g., engineering works, dredging and dumping activities, and land reclamations)^12–14^. Such anthropogenic interventions drastically modify sediment budgets in estuarine and deltaic systems, having important consequences for navigation, ecology, and flood safety. Moreover, current sediment management policies may impact the system's capacity to cope with sea-level rise in the coming decades^15^.

In situ measurements, remote satellite observations, and numerical simulations have shown that estuary sediment fluxes are driven by the combination of hydro-meteorological forcing, such as tides, waves, wind, and river discharge^16–20^. These fluxes directly depend on the availability of sediment pools originating from marine, estuarine and riverine sources^2^. The export of estuarine and riverine sediments to coastal seas is usually associated with wave-induced sediment resuspension, whereas the import of marine sediment within estuaries mainly results from tidal and gravitational circulations^18^. Nonetheless, the contribution of gravitational circulation to sediment import strongly depends on the hydrological cycle and may differ from an estuary to another^20–22^. In addition, it remains difficult to relate sediment fluxes to external forcing due to the general concomitance of antagonist meteorological events, such as stormy (i.e., high waves) and wet (i.e., high river discharge) events concurrently occurring during North-Atlantic winter seasons.

Net sediment transfers between rivers and seas depend on the estuary hydrological and hydrodynamic regimes, which are modulated by the estuary morphology and the sediment availability^23^. Human activities can drastically change the upstream river supplies (e.g., through dam construction^24^), the local sediment nature (e.g., through dredging activities^25^), and the estuary morphology (e.g., through harbor extension and channelization^26^). Guo et al.^27^ recently investigated a centennial hydro-morphodynamic evolution of the Changjiang Estuary (China) to highlight the influence of anthropogenic pressures on estuary sediment import–export. More specifically, they observed that a narrower funnel-shaped estuary resulting from intensive human activities induced a shift from an ebb- to flood-dominated estuary, leading to increased sediment import and channel aggradation. Such behavior was observed as well in estuaries following severe channel deepening, shifting systems from normal to hyper-turbid states^28,29^. Contrastingly, Cox et al.^15^ observed a negative sediment budget (i.e., sediment export) in the Rhine-Meuse Delta (The Netherlands) since the 1980s, resulting from engineering works and dredging activities. Nonetheless, some estuaries can keep balanced sediment budgets over hundreds of years despite dredging activities, such as the Humber Estuary (UK)^30^.

In addition to anthropogenic pressures, meteorological changes can impact sediment budgets in modulating estuarine forcing (e.g., river discharge and storminess) and exacerbating drastic perturbations as extreme events^31–34^. In the context of global changes, it is necessary to disentangle the effects of meteorological and human-induced changes on estuarine fluxes for better understanding and predicting particulate transfers along the land-sea continuum. Therefore, this study aims at investigating the relative contributions of key forcing processes on net estuary sediment fluxes. It focuses on sediment transfers between a macrotidal estuary and its adjacent coastal sea under different anthropogenic and meteorological pressures.

**Figure 1:**
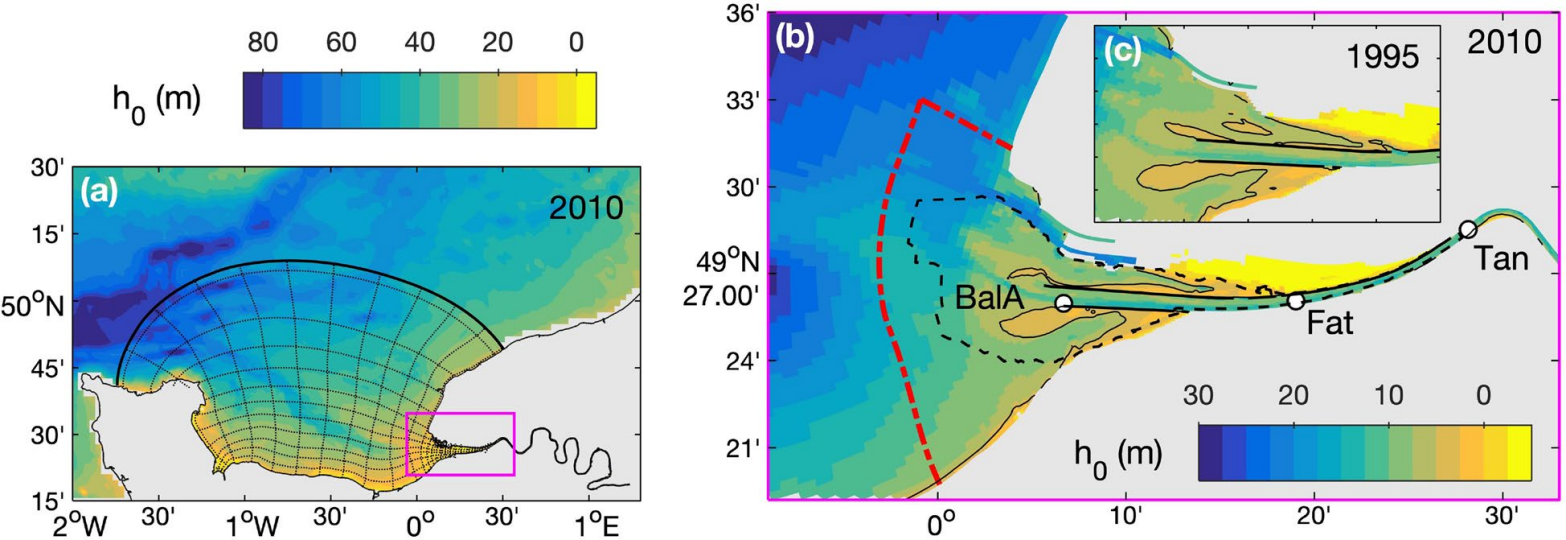
Bathymetry of the Seine Estuary, NW France (with *h*_0_ the water depth relative to mean sea level). (**a**) Full domain of the MARS3D model with every tenth grid cells represented, (**b**) focus on the lower estuary in 2010, and (**c**) focus on the estuary mouth in 1995. In panels (**b**, **c**), solid black contours represent 5-m isobaths, characterizing intertidal areas. In panel (**b**), the black dashed contour represents the comparison area between field surveys and numerical simulations, the red dash-dot line represents the estuary-sea boundary where sediment fluxes are computed, and the white circles represent Balise A, Fatouville, and Tancarville locations (‘BalA’, ‘Fat’, and ‘Tan’, respectively).

The analysis is based on a 22-year numerical hindcast of the Seine Estuary (France) comparing two periods (1990–2000 and 2005–2015) with contrasted human-altered morphologies (i.e., a deeper and narrower estuary in 2010 than in 1995; Fig. 1). The influence of meteorological changes on sediment transfers is investigated through a global analysis of mean differences over the two periods, but we do not specifically analyze individual extreme events, as already examined for severe tropical storms^35,36^. Although sediment import–export can depend on the occurrence between tidal phasing and meteorological forcing^17^, this work focuses on fortnightly tide-averaged fluxes to draw a conceptual pattern of wave-river discharge contributions to sediment transfers between estuaries and seas.

## Results and discussion

### Changes in forcing and environmental parameters

**Figure 2:**
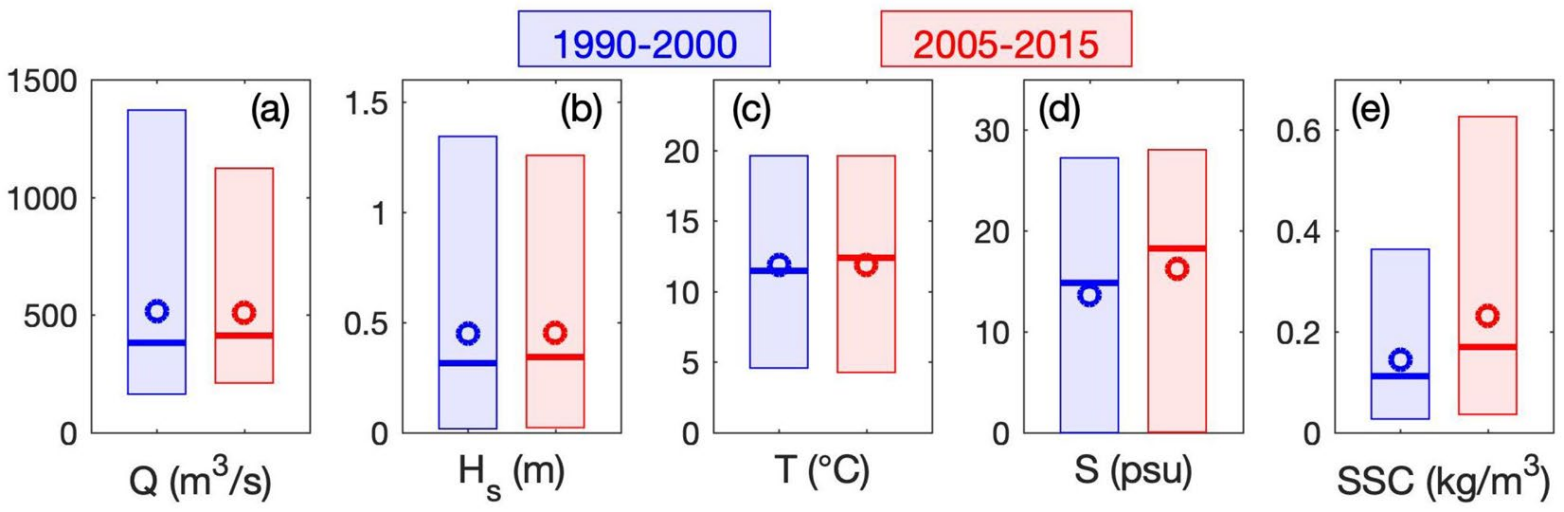
Comparison of characteristic environmental parameters between 1990–2000 (blue) and 2005–2015 (red): (**a**) river discharge *Q*, (**b**) significant wave height *H*_*s*_ at the estuary-sea boundary (red dash-dot line in Fig. 1b), (**c**–**e**) near-bed temperature *T*, salinity *S* and *SSC*, respectively, at Fatouville (‘Fat’ in Fig. 1b). Boxes range from 5 to 95th percentiles; thick lines and circles represent median and mean values, respectively.

Changes in meteorological forcing during the last decades are analyzed through the median and extreme values (i.e., 5th, 50th, and 95th percentiles) over the two investigated periods (i.e., 1990–2000 and 2005–2015), as illustrated in Fig. 2. Statistics on the river discharge *Q* are based on the Seine and its tributaries and statistics on the significant wave height *H*_*s*_ are computed at the estuary-sea boundary (red dash-dot line in Fig. 1b). River discharge and wave forcing present similar variabilities with an increase of median values (*p*_*50*_: + 8% and + 9%, respectively) and a decrease of the extreme values (*p*_*95*_: − 18% and − 6%, respectively). Such changes are not reflective of climate-induced changes (i.e., increased extreme events and reduced mean river discharge^31–33^). However, these forcing conditions are representative of natural variability during two contrasted meteorological decades (Supplementary Figure S1).

Figure 2c–e illustrates the simulated mean changes in dominant environmental parameters—as near-bed temperature *T*, salinity *S*, and suspended sediment concentration *SSC*—within the central salt wedge and ETM areas (i.e., at Fatouville in Fig. 1b). Note that this location is representative of changes occurring in the lower estuary (Supplementary Figures S2–S5). The median temperature increased by 1 °C (+ 8%), whereas the mean temperature only increased by 0.2 °C. These changes are in agreement with observations of global warming in the English Channel^37^. The difference between median and mean values highlights changes in temperature distributions, but it also alerts us on the estimate sensitivity to statistic computations. Despite an increased river discharge, the median salinity substantially increased (*p*_*50*_: + 3.4 psu, + 23%), with a moderate increase of extreme values. These changes mainly result from the density-induced salinity intrusion enhanced by anthropogenic changes (i.e., channel deepening and estuary narrowing), as observed by Grasso and Le Hir^26^. Finally, changes in SSC are even stronger, both in median and extreme values (*p*_*50*_: + 0.06 kg/m^3^, + 52%; *p*_*95*_: + 0.26 kg/m^3^, + 72%). As observed for salinity changes, the SSC increase mainly results from the estuary deepening and narrowing^26^, which increases tide- and density-induced upstream sediment transport and potentially shifts systems toward hyper-turbid states^9,38,39^. In similar small, narrow, and converging estuaries (e.g., the Ems and the Loire estuaries), Winterwerp et al.^29^ observed that the shift from normal to hyper-turbid states was related to both engineering works (i.e., estuary narrowing and deepening) and hydraulic drag reduction. However, the data suggest that the development of hyper-turbid conditions upon passing a tipping point may take one to two decades.

### Comparison of annual sediment fluxes between 1990–2000 and 2005–2015

**Figure 3:**
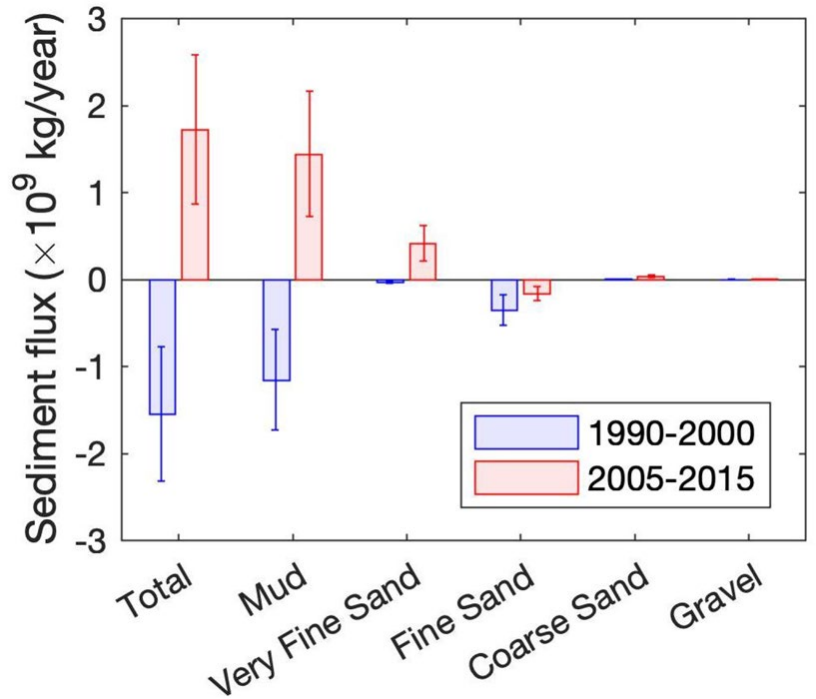
Comparison of yearly-averaged sediment fluxes at the estuary-sea boundary (red dash-dot line in Fig. 1b) between 1990–2000 (blue) and 2005–2015 (red), for each sediment class and the sum (Total). Positive fluxes are directed up-estuary (i.e., import) and negative fluxes are directed seaward (i.e., export). Brackets represent inter-annual standard deviations.

At the annual time scale, total sediment fluxes present contrasted behaviors along the two periods (Fig. 3), with a net export of estuarine and riverine sediments in 1990–2000 (− 1.55 × 10^9^ kg/year) and a net import of marine sediment in 2005–2015 (+ 1.72 × 10^9^ kg/year). Estuary mouth sediment volumes measured during the 1990–2020 period corroborate these simulations, with a decreasing trend before 2005 and a net increase after 2005 (Supplementary Figure S6). These changes mainly result from the mud dynamics, representing 75% and 84% of the total fluxes in 1990–2000 and 2005–2015, respectively. The rest of the changes are attributed to very fine and fine sands, as coarser sediments (i.e., coarse sand and gravel) contribute less than 3% of the total fluxes. Note that these coarse sediments (*d* > 800 µm) are mainly imported within the estuary, in contrast with the Humber Estuary where Townend and Whitehead^30^ identified a net export of coarse sediment. Fine sand (210 µm) is exported from the Seine Estuary, whilst mud changes from export to import during the time frame.

The shift from total sediment export to import has also happened in other systems (e.g., Changjiang Estuary^27^) and can be the result of bathymetric changes (estuary deepening and narrowing; Fig. 1b,c, Supplementary Figure S2). Nonetheless, these changes may also be the response to meteorological forcing, in particular, changing river discharge and wave forcing (Fig. 2a,b).

### Sediment flux response to meteorological forcing

**Figure 4:**
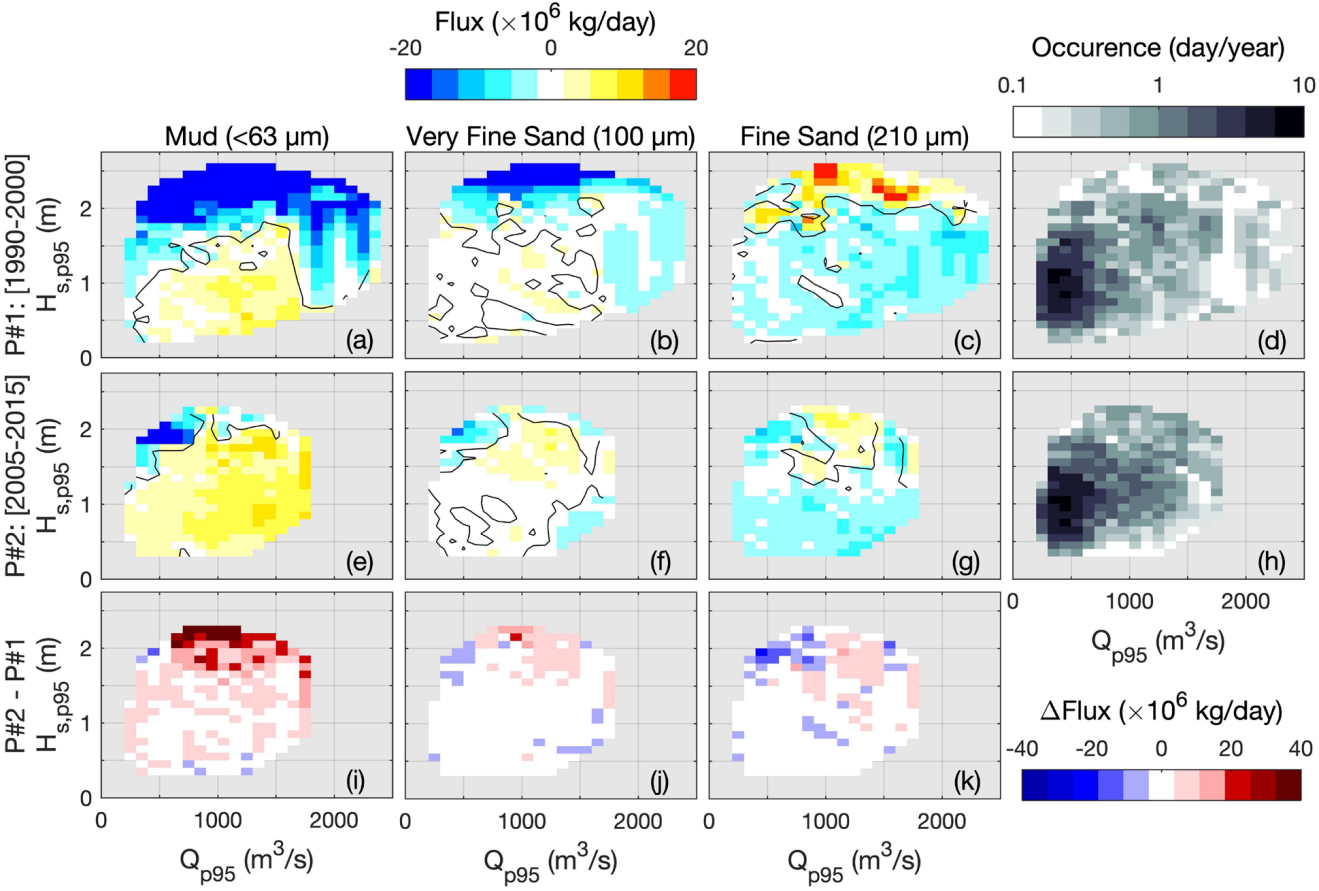
Comparison of fortnightly-averaged sediment fluxes at the estuary-sea boundary (red dash-dot line in Fig. 1b) between (top panels) the first period P#1 [1990–2000] and (middle panels) the 2nd period P#2 [2005–2015], in function of the fortnightly-95th percentiles of river discharge (*Q*_*p95*_) and significant wave height (*H*_*s,p95*_) forcing, for the three dominant sediment classes (**a**, **e**) mud, (**b**, **f**) very fine sand and (**c**, **g**) fine sand. Positive fluxes are directed up-estuary (i.e., import) and negative fluxes are directed seaward (i.e., export). Bottom panels (**i**–**k**) represent the flux differences *∆Flux* between P#2 and P#1. Panels d and h represent the occurrence of *Q*-*H*_*s*_ forcing in 1990–2000 and 2005–2015, respectively.

To unravel the relative contributions of meteorological forcing (i.e., river discharge and wave conditions) on sediment transfers, sediment fluxes are computed at a shorter time scale. We used a fortnightly sliding window to average sediment fluxes, river discharge, and wave forcing. The 95th percentiles of river discharge and significant wave height are used to represent the forcing parameters over the fortnightly periods because they showed greater correlations with sediment fluxes rather than median or mean values. This is mainly explained by the large contribution of intense events to mean changes in SSC and sediment fluxes, as high river discharge events^35,36^. Net fluxes are analyzed through a *Q*-*H*_*s*_ diagram for the dominant sediment classes (i.e., mud, very fine and fine sands) and the two periods (Fig. 4). Sediment fluxes are averaged over *Q* and *H*_*s*_ bins with a spacing of 100 m^3^/s and 0.1 m, respectively. The corresponding occurrences (Fig. 4c,h) illustrate that the 1990–2000 period experienced stronger conditions both in river discharge and wave forcing than the 2005–2015 period (as observed in Fig. 2a,b).

In 1990–2000, the mud fluxes present a clear pattern with export increasing due to wave conditions (Fig. 4a), resulting from the increased sediment resuspension^17,18^. Interestingly, the mud export decreases when river discharge increases and even turns to import for moderate to large river discharges (i.e., from 400 to 1500 m^3^/s). This is characteristic of the enhanced gravitational circulation simulated by Schulz et al.^20^ in the Seine Estuary. Furthermore, it is in agreement with the observations of Sommerfield and Wong^22^ in the Delaware Estuary (USA), highlighting that the estuary has a large capacity to buffer large river discharge and suppress the export of suspended sediment to the Delaware Bay. Nevertheless, mud fluxes can export again for high river discharges (i.e., > 1500 m^3^/s) when the density-induced import at the bottom is not sufficiently strong to compensate for the large sediment export at the surface.

It is remarkable to observe that sands present opposite behaviors depending on size. There is a tendency to export very fine sand (100 µm), similarly to mud but associated with weaker fluxes, but import fine sand (210 µm), when wave conditions are the strongest (Fig. 4b,c). Such behaviors result from different erodibility thresholds and suspension durations associated with subtidal currents (i.e., ebb-flood asymmetries in both current intensity and duration; Nidzieko^40^). These results point out that different sand classes need to be considered for properly simulating the diversity of natural sand fluxes and the resulting morphological evolutions.

Sediment fluxes substantially changed in 2005–2015 with more import of mud and very fine sand, but less import of fine sand (Fig. 4e–g). Such differences can be related to changes in both meteorological and anthropogenic pressures, which are specifically investigated in the following section.

### Untangling the influences of meteorological and anthropogenic changes on mud fluxes

**Figure 5:**
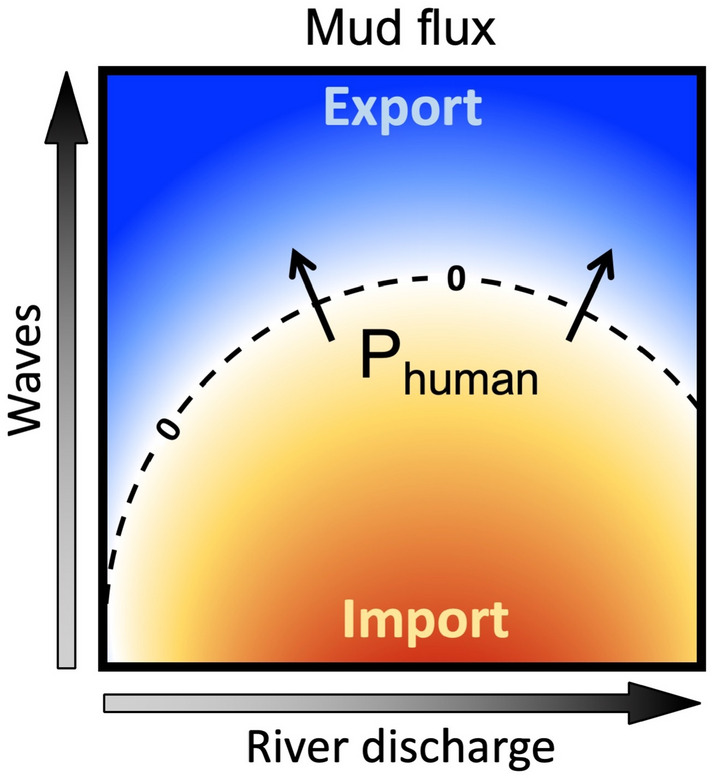
Schematic of mud fluxes in function of river discharge and wave forcing. Warm colors represent up-estuary fluxes (i.e., import) and cool colors represent seaward fluxes (i.e., export). *P*_*human*_ denotes the human-induced pressures impacting the diagram isolines.

Mud fluxes represent more than 75% of the total sediment fluxes between the estuary and the coastal sea and these very fine particles contribute to biogeochemical processes along the land-sea continuum (e.g., adsorption and desorption mechanisms). Therefore, the present section focuses on the sensitivity of mud transfers to meteorological and anthropogenic changes. Over the 1990–2000 period, results highlighted that mud export increases with wave forcing, but moderate to large river discharges support mud import (Fig. 4a). This pattern can be schematized through the *Q*-*H*_*s*_ diagram in Fig. 5. Changes in meteorological conditions between 1990–2000 and 2005–2015 are observed throughout changes in *Q*-*H*_*s*_ occurrences (Fig. 4d,e). For instance, the milder conditions experienced in 2005–2015 limit the mud export occurring for large river discharge and wave events, and thus favor mud import within the estuary.

Within the same *Q* and *H*_*s*_ ranges, i.e., for the same meteorological conditions, the mud flux pattern changes between the two periods (Fig. 4a,e). For instance, the isoline delimiting mud import–export at *Q* = 1000 m^3^/s is close to *H*_*s*_ = 1.5 m in 1990–2000 and rises around *H*_*s*_ = 2 m in 2005–2015. These changes in mud flux contours are illustrated through the positive flux difference in Fig. 4i (i.e., 2nd period minus 1st period), characterizing more import (or less export) of mud in 2005–2015 than in 1990–2000. Such behavior can be attributed to human-induced changes, which impacted the system functioning via the estuary deepening and narrowing, as observed by Guo et al.^27^. It is also supported by Grasso and Le Hir^26^ who simulated an intensification of density-induced circulation due to bathymetric changes in the Seine Estuary from 1960 to 2010. Thus, anthropogenic pressures (*P*_*human*_) would affect the mud pattern schematized in Fig. 5 by shifting the *Q*-*H*_*s*_ diagram isolines. In other words, the mud fluxes would respond differently to similar meteorological forcing due to human-induced morphological changes.

**Figure 6:**
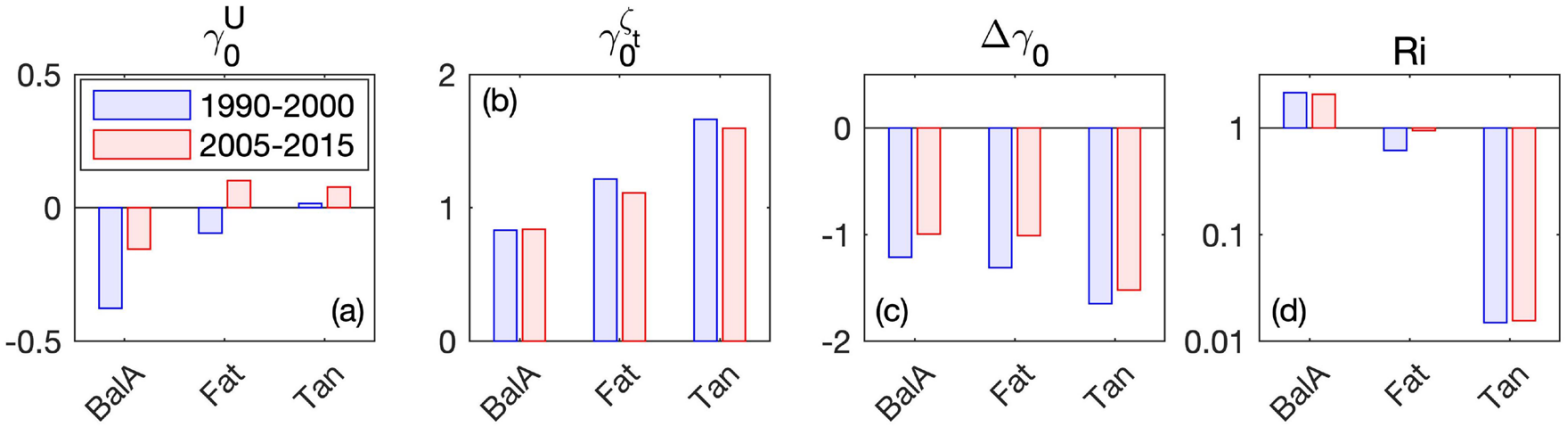
Comparison of yearly-averaged hydrodynamic parameters at different stations along the estuary (‘BalA’, ‘Fat’, and ‘Tan’ in Fig. 1b) between 1990–2000 (blue) and 2005–2015 (red): (**a**) tidal velocity skewness $\gamma_{0}^{U}$, (**b**) tidal duration asymmetry $\gamma_{0}^{{\zeta_{t} }}$, (**c**) difference between tidal velocity skewness and duration asymmetry $\Delta \gamma_{0}$, and (**d**) vertical Richardson number *Ri*.

To further investigate the underlying processes responsible for sediment transport changes between the two periods, Fig. 6 illustrates hydrodynamic parameters representative of tidal asymmetries and water density gradients (Eqs. 2–5) at three stations along the lower estuary (Balise A, Fatouville, and Tancarville; Fig. 1b). Both tidal velocity skewness ${\varvec{\gamma}}_{0}^{{\varvec{U}}}$ and duration asymmetry ${\varvec{\gamma}}_{0}^{{{\varvec{\zeta}}_{{\varvec{t}}} }}$ increased up-estuary, as tide became more distorted. However, from 1990–2000 to 2005–2015, velocity skew increased (i.e., more flood dominant or less ebb dominant) and duration asymmetry decreased (i.e., shorter falling water). It resulted in a larger ${{\varvec{\Delta}}}{\varvec{\gamma}}_{0}$ in 2005–2015 (i.e., less negative), corresponding to a reduction of the enhanced velocity ebb dominance. The vertical Richardson number *Ri* decreased up-estuary, as mixing became more pronounced and overwhelmed stratification. *Ri* did not significantly change at Balise A and Tancarville between 1990–2000 and 2005–2015; however, it substantially increased at Fatouville, characterizing an enhanced stratification in the central part of the ETM^6^. Both changes in tidal asymmetries and density gradients support an increased sediment transport up-estuary and explain the shift from an exporting to an importing system. This agrees with studies observing shifts from normal to hyper-turbid estuaries following morphological changes as deepening and narrowing^28,29,38^.

## Conclusions

A 22-year numerical hindcast (1990–2000 and 2005–2015) of the Seine Estuary sediment dynamics has been analyzed to investigate the relative contributions of meteorological and anthropogenic changes on sediment import–export between the estuary and its adjacent coastal sea. From 1990–2000 to 2005–2015, human pressures induced substantial morphological changes leading to a deeper and narrower estuary; meteorological conditions (i.e., river discharge and wave forcing) changed with larger median conditions but smaller extreme events. These changes resulted in increasing salinity intrusion and SSC within the estuary.

Net sediment fluxes at the estuary-sea boundary are related to river discharge and wave forcing. Increasing wave conditions enhance the export of very fine sediments (≤ 100 µm) and import of coarser sediments (≥ 210 µm). Although intense river discharges induce mud export to the sea, moderate to large river discharge conditions support very fine sediment import. The reduction of extreme conditions in the most recent period (2005–2015) reduces mud export to the coastal sea. In addition, human-induced morphological changes perturbated the estuary sediment dynamics and enhanced mud import. Consequently, in less than 25 years, meteorological and anthropogenic changes shifted the estuary from an exporting to an importing system.

The mud flux response to meteorological and anthropogenic changes is schematized through a “river discharge-wave diagram” where meteorological conditions determine the estuary forcing, and human pressures affect the system's functioning. Such a schematic has to be challenged over other tidal estuaries. Nevertheless, it represents an excellent tool to investigate potential trajectories in estuary sediment import–export, directly impacting other compartments of the estuarine ecosystem (e.g., biogeochemistry, biology, and ecology).

## Methods

### Study area

The Seine Estuary (NW France) is a semidiurnal macrotidal system with a tidal range varying from 3 to 8 m at the estuary mouth. It is one of the largest estuaries on the Northwestern European continental shelf and stretches from the Bay of Seine open to the English Channel to the weir of Poses upstream, the tidal influence limit (Fig. 1). The Seine River discharge ranges from 100 to 2300 m^3^/s with a mean annual discharge around 450 m^3^/s and a mean river sediment supply around 0.7 × 10^9^ kg/year^20,41^.

The funnel-shaped estuary is exposed to western winds so that the intertidal regions at the mouth are subject to erosion under the combined effect of waves and currents^42,43^. Waves enter the bay from the northwest with typical significant wave heights of 0.5 m and peaks of more than 3.5 m in front of the estuary mouth. It is characterized by the presence of an ETM that has a pronounced control on the sedimentation patterns of subtidal areas and intertidal mudflats from the estuary mouth up to the upstream freshwater limit, which is few kilometers upstream of Tancarville (‘Tan’ in Fig. 1b)^6,44–46^.

During the last century, the Seine Estuary has been vastly altered by human activity^44^. As a result, it was changed from a dominantly natural system to a human-controlled system^26^. In the last decades, i.e., from the 1990s to the 2010s, extensive engineering works induced a deepening and narrowing of the lower estuary. It mainly resulted from the large extension of the Grand Port Maritime du Havre (GPMH) at the estuary mouth (named as “*Port 2000*”) and the main channel deepening and dredging to access the Grand Port Maritime de Rouen (GPMR) approximately 120 km upstream of the mouth (Fig. 1b,c). Changes in dredging activities resulted in a deeper navigation channel (around 1–2 m) after 2005 (Supplementary Figure S2).

### Numerical model set-up

The ARES hindcast simulations are based on the process-based hydrodynamic and sediment dynamic model developed and validated by Grasso et al.^6^. This model has been used by Schulz et al.^20^ to investigate sediment response to idealized hydro-meteorological forcing and by Grasso and Le Hir^26^ to investigate the influence of contrasted morphologies on ETM dynamics. The model set-up is extensively detailed in the above-mentioned studies; nonetheless, the main model characteristics are outlined below.

A non-orthogonal curvilinear mesh extends from the Bay of the Seine to the weir at Poses (Fig. 1a) with a resolution around 30 × 100 m^2^ in the lower estuary (i.e., from the mouth to Tancarville; Fig. 1b), corresponding to the main ETM excursion area. The hydrodynamic model is based on the hydrostatic model MARS3D^47^ discretized with 10 equidistant sigma layers. The circulation model is forced by the main tidal components at the sea boundary (CST France, SHOM), the wind stresses and pressure gradients provided by the meteorological ARPEGE model (Meteo-France), and the measured daily discharges from the Seine River and its tributaries. Waves are simulated from the WAVEWATCH III® model^48^ based on a series of embedded computational grids, from a large-scale model of the Atlantic Ocean down to a local model with the same resolution as the circulation model.

The hydrodynamic model is coupled with the MUSTANG sediment model for cohesive and non-cohesive mixtures^49–51^. This multi-layer model accounts for the spatial and temporal variations of sand and mud content in the sediment, as well as for consolidation processes, and resolves advection/diffusion equations for different classes of particles in the water column. This model considers five classes of sediment representative of the Seine Estuary sediment modes^52^: one gravel (diameter *d* = 5 mm), three sands (coarse: *d* = 800 µm, fine: *d* = 210 µm, and very fine: *d* = 100 µm) and one mud. Sediment is initially distributed over a 1-m thick bed according to a realistic bed coverage^52^. The mud advection is calculated using a complete 3D scheme with a variable settling velocity accounting for flocculation processes^53^. The riverine sediment supplies (defined as mud) are imposed at the river discharge locations and vary with the freshwater discharges^41^. In addition, the model simulates the dredging and dumping activities related to the maintenance strategy of the GPMH and GPMR access channels. This human-induced sediment transfer is simulated following the method detailed by Grasso et al.^6^. The upper sediment layers in the dredged areas are removed if the sediment deposit exceeds a prescribed base elevation, then the dredged sediment mass is released in the lowest cells of the water columns in the dumping areas.

Hindcast simulations over the 1990–2000 and 2005–2015 periods were run through independent years following a morphostatic approach (i.e., no morphodynamic coupling), which is relevant for analyzing sediment dynamics at time scales of few years (< 5–10 years) when morphological changes remain relatively small to hydrodynamic processes. The 1995 and 2010 bathymetries were used to simulate the 1990–2000 and 2005–2015 hindcast, respectively. Each year was run twice to consider a 1-year spin-up period before analyzing the half-hourly outputs^6,20,26^. Moreover, simulations ran from October to October to respect annual hydrological cycles and not to cut down wet and dry periods.

### Validation of sediment budgets and fluxes

**Figure 7:**
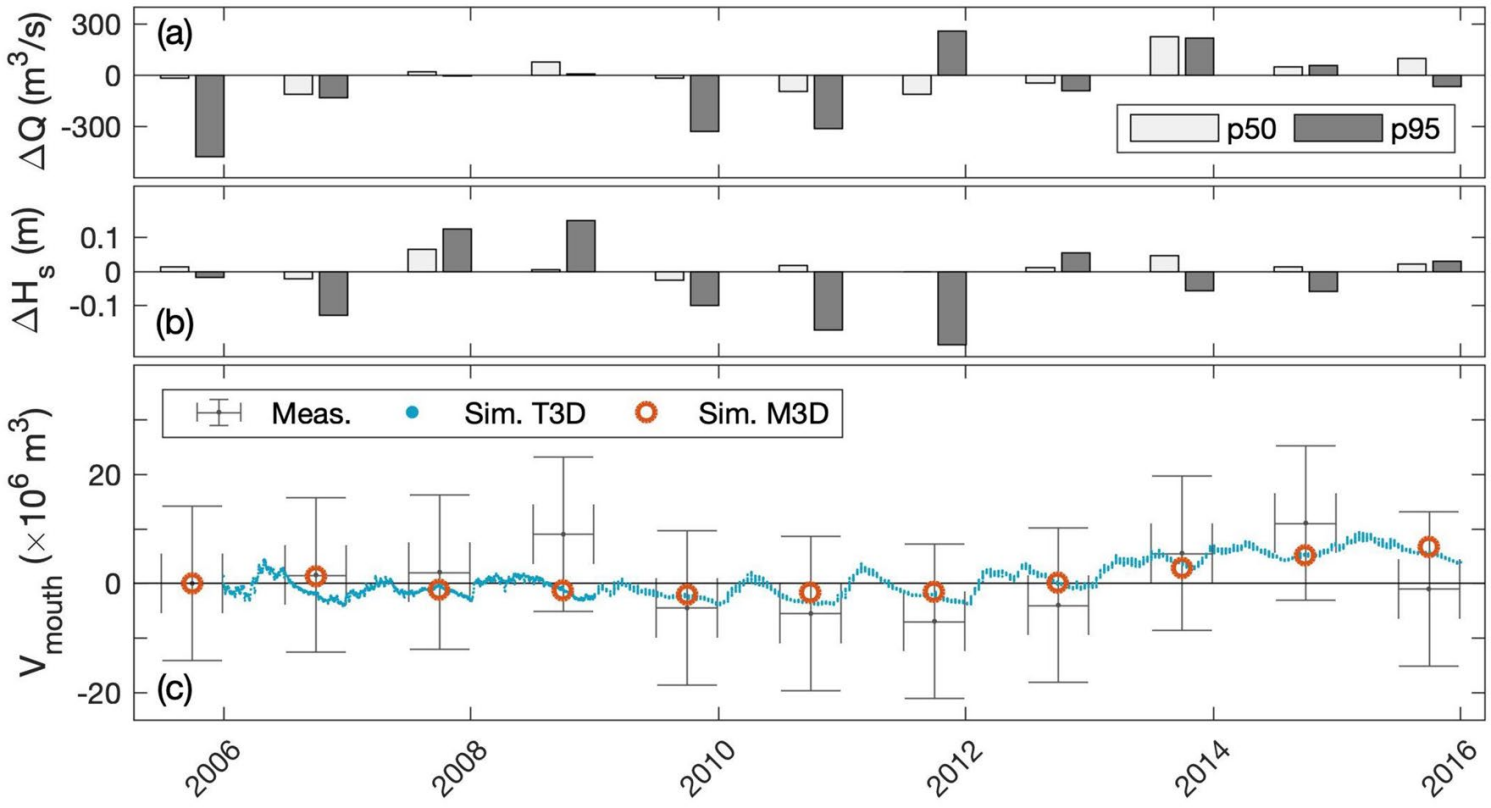
Annual anomalies from 2005 to 2015 of 50th (white) and 95th (gray) percentiles in (**a**) river discharge *∆Q* and (**b**) significant wave height *∆H*_*s*_. (**c**) Sediment volume *V*_*mouth*_ in the estuary mouth (black dashed contour in Fig. 1b), measured from bathymetric surveys (gray brackets), and simulated from the morphodynamic model TELEMAC3D ‘T3D’ from ARTELIA (blue dots) and the morphostatic model MARS3D ‘M3D’ used in this study (brown circles).

Simulations of sediment transfers between estuaries and coastal seas are prone to large uncertainties associated with both validation dataset and numerical model parameterization^54^. Grasso et al.^6^ validated the Seine Estuary model in terms of hydrodynamics, salinity, and SSC from tidal to annual time scales at different stations within the estuary (Supplementary Figures S7–S9). However, Ganju and Schoellhamer^21^ recommend using bathymetric surveys for evaluating the capabilities of a model to properly reproduce sediment budgets and fluxes. Therefore, the model simulations were compared to annual bathymetric changes measured in the lower estuary by the GPMR (black dashed contour in Fig. 1b) during the second period (2005–2015, Fig. 7c), with regard to annual anomalies of river discharge and wave forcing (Fig. 7a,b). The large uncertainties associated with bathymetric changes are due to both the vertical uncertainties of bathymetric surveys (± 0.1 m) and the timeframe to cover the entire estuary mouth (~ 6 months). Thus, these measurements have to be considered as a qualitative view of sediment volume changes in the estuary mouth. In addition, these large uncertainties inform us that: (1) errors on “ground-truth” measurements can be very large; and (2) field measurements are still needed to more accurately assess estuarine morphological changes.

The present simulations result from morphostatic modeling, so no bathymetric changes in the hydrodynamic model are computed. However, the bed sediment thickness can change with erosion, deposition, and consolidation processes. Hence, sediment volume changes can be computed from differences in bed thickness over the same area as the GPMR bathymetric surveys. While the simulations do not exactly match the measurements, they prove to be in a good capacity to reproduce the main volume changes observed in the estuary mouth over 11 years.

To extend the validation, the simulated volume changes from our MARS3D ‘M3D’ model are compared to volume changes resulting from morphodynamic modeling carried out by ARTELIA, based on the finite element TELEMAC3D ‘T3D’ model^55^. T3D continuously simulated ten years, starting from the 2006 bathymetry and with bathymetric adjustment via morphodynamics coupling, whereas M3D simulated 11 independent years considering the 2010 bathymetry. The interest in such a model intercomparison is twofold: (1) both models present very similar results although hydrodynamics and sediment dynamics are differently parameterized and resolved, which provides confidence in the simulation reliability; and (2) the morphostatic modeling ‘M3D’ used in this study is shown to be relevant for investigating sediment volume changes up to 5 years around a given bathymetry.

In addition, bathymetric surveys recorded over a smaller area at the estuary mouth provide information on sediment dynamics over the 1990–2020 period (Supplementary Figure S6). The observations of sediment volume changes reveal a decreasing trend before 2005 and a net increase after 2005, which is relatively well captured by the model. Therefore, the capacity to properly simulate changes in sediment volumes provides confidence in the ability to simulate sediment budgets and fluxes. However, changes in sediment volumes do not exactly correspond to changes in sediment mass. For instance, consolidation processes induce a decrease in sediment volume (i.e., sediment compaction), but the sediment mass does not change^50^. Moreover, changes in sediment porosity due to changes in mud-sand mixtures affect the bed volume and not the mass^56^. Thus, while bathymetric surveys are limited to analyze sediment budgets and fluxes, simulations provide adapted knowledge as changes in sediment mass are explicitly computed.

### Sediment flux computation

Sediment fluxes along the Seine Estuary result from a balance between the Seine River sediment supply, the estuarine sediment, and the marine sediment in the Bay of Seine. In this study, the net sediment fluxes are computed at the estuary-sea boundary (red dash-dot line in Fig. 1b), in agreement with the ‘offshore’ boundary used by Schulz et al.^20^. It represents a suitable limit beyond which seaside morphological changes are small compared to estuarine changes^20,26^ (Supplementary Figure S2) and properly characterizing sediment transfers between the estuary and the bay. This boundary definition implies that net fluxes result from exchange between (1) marine sources seaward of the boundary and (2) estuarine and riverine sources upward of the boundary. Thus, import is associated with marine sediment advected up-estuary, and export is associated with estuarine and riverine sediments advected seaward.

The fluxes *F*_*i,∆t*_ during a period *∆t* are computed for each sediment class *i* as the difference in sediment mass *M*_*i*_ (i.e., sediment budget) in the lower estuary area, which is defined between the estuary-sea boundary and Tancarville (Fig. 1b), and considering the incoming sediment fluxes at Tancarville *F*_*i,Tan*_:

1 $$ F_{i,\Delta t} = \Delta M_{i,\Delta t} + \mathop \smallint \limits_{0}^{\Delta t} F_{i,Tan} dt $$

with a positive flux oriented up-estuary (i.e., import) and a negative flux oriented seaward (i.e., export). *F*_*i,Tan*_ is integrated online at every time step across the channel section^20^ and *M*_*i*_ is the sum of sediment masses in both water and bed compartments.

### Hydrodynamic parameter computation

As it propagates up-estuary, the tide is distorted and becomes more and more asymmetric; it induces a tidal pumping mechanism that can transport sediment up-estuary^4^. Different proxies can be used to characterize the tidal asymmetry^57,58^, but Nidzieko^40^ suggested that quantifying tidal asymmetry via skewness should be preferred over traditional metrics in estuaries with mixed tides. Following Nidzieko and Ralston^59^, it reads:

2 $$ \gamma_{0} = \frac{{\mu^{3} }}{{\mu_{2}^{3/2} }}, $$

where the *m*th moment about zero is defined as

3 $$ \mu_{m} = \frac{1}{n - 1}\mathop \sum \limits_{i = 1}^{n} \left( {x_{i} } \right)^{m} $$

and *n* is the number of samples *x*_*i*_. In this study, we use this method to quantify:

1. The ebb-flood tidal current asymmetry $\gamma_{0}^{U}$, referred to as the ‘velocity skewness’, based on the bottom velocity *U* and quantified by substituting *x* = *U*;
2. The tidal duration asymmetry in the rise and fall of water level $\gamma_{0}^{{\zeta_{t} }}$, referred to as the ‘duration asymmetry’. It is quantified by substituting the time derivative $x = \zeta_{t} = \partial \zeta /\partial t$.

For velocity, the tide is ebb dominant for $\gamma_{0}^{U} < 0$ and flood dominant for $\gamma_{0}^{U} > 0$; the duration of falling water is shorter than rising water for $\gamma_{0}^{{\zeta_{t} }} < 0$ and longer for $\gamma_{0}^{{\zeta_{t} }} > 0$. In addition, differences between velocity skewness and duration asymmetry

4 $$ \Delta \gamma_{0} = \gamma_{0}^{U} - \gamma_{0}^{{\zeta_{t} }} $$

can be diagnostic of how tides are manifest as currents on the tidal flat. Negative $\Delta \gamma_{0}$ indicates enhanced velocity ebb dominance (or less flood-dominant velocities depending on the signs of $\gamma_{0}^{U}$ and $\gamma_{0}^{{\zeta_{t} }}$) relative to the rise/fall asymmetry in the sea surface.

As tidal velocity skewness and duration asymmetry are good proxies of tidal pumping, the vertical Richardson number is representative of the density-induced circulation^59–62^. It is expressed as:

5 $$ Ri = N^{2} /S^{2} $$

where $N^{2} = \frac{g}{{\rho_{0} }}\frac{\partial \rho }{{\partial z}}$ is the Brunt–Väisälä frequency, *g* is the gravity acceleration, *ρ* is the water density (with the reference *ρ*_*0*_), and $S = \frac{\partial u}{{\partial z}}$ is the vertical shear of horizontal velocity. Miles^63^ suggests the existence of a critical *Ri* value of 0.25 above which a stable salinity stratification tends to occur, while below which the stratification tends to be unstable and hence tidal mixing is likely to occur. These parameters were computed at three stations along the estuary (i.e., Balise A, Fatouville, and Tancarville in Fig. 1b) over a 14-day sliding window and averaged over the two investigated periods (i.e., 1990–2000 and 2005–2015).

## Supplementary Information

Supplementary Information.
