# Supplementary material for: Unraveling the impacts of meteorological and anthropogenic changes on sediment fluxes along an estuary-sea continuum
Source: Sci Rep. 2021 Oct 12;11:20230. doi: 10.1038/s41598-021-99502-7 (PMC8511176; doi:10.1038/s41598-021-99502-7)
Supplement: Supplementary file 1 — Supplementary Information. [file 41598_2021_99502_MOESM1_ESM.pdf]

Unraveling the impacts of meteorological and anthropogenic changes on sediment fluxes along an estuary-sea continuum: Supplementary information

Florent Grasso[1,*], Eliott Bismuth[1], and Romaric Verney[1]

[1]Ifremer – DYNECO/DHYSED, Centre de Bretagne, CS 10070, Plouzané, F-29280, France
*florent.grasso@ifremer.fr

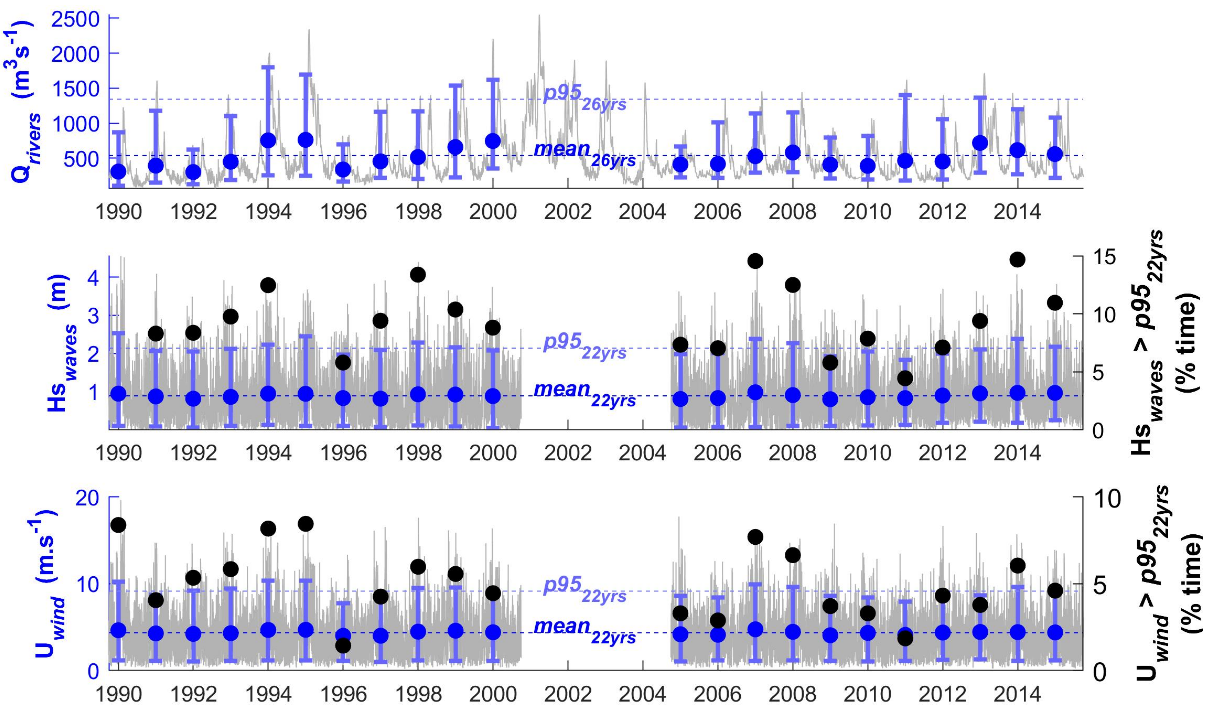

Figure S1. Main forcing conditions of the Seine Estuary between 1990 and 2015. (Top panel) River discharge of the Seine River and its tributaries Q_{rivers}, (middle panel) significant wave height at the estuary mouth Hs_{waves}, and (bottom panel) mean wind velocity over the estuary domain U_{wind}. Gray lines represent high-frequency data, blue dots represent mean values with whiskers representing the 5th and 95th percentiles, and black dots represent the time (in % of year) when conditions exceed the 95th percentile value calculated over the 1990-2015 period.

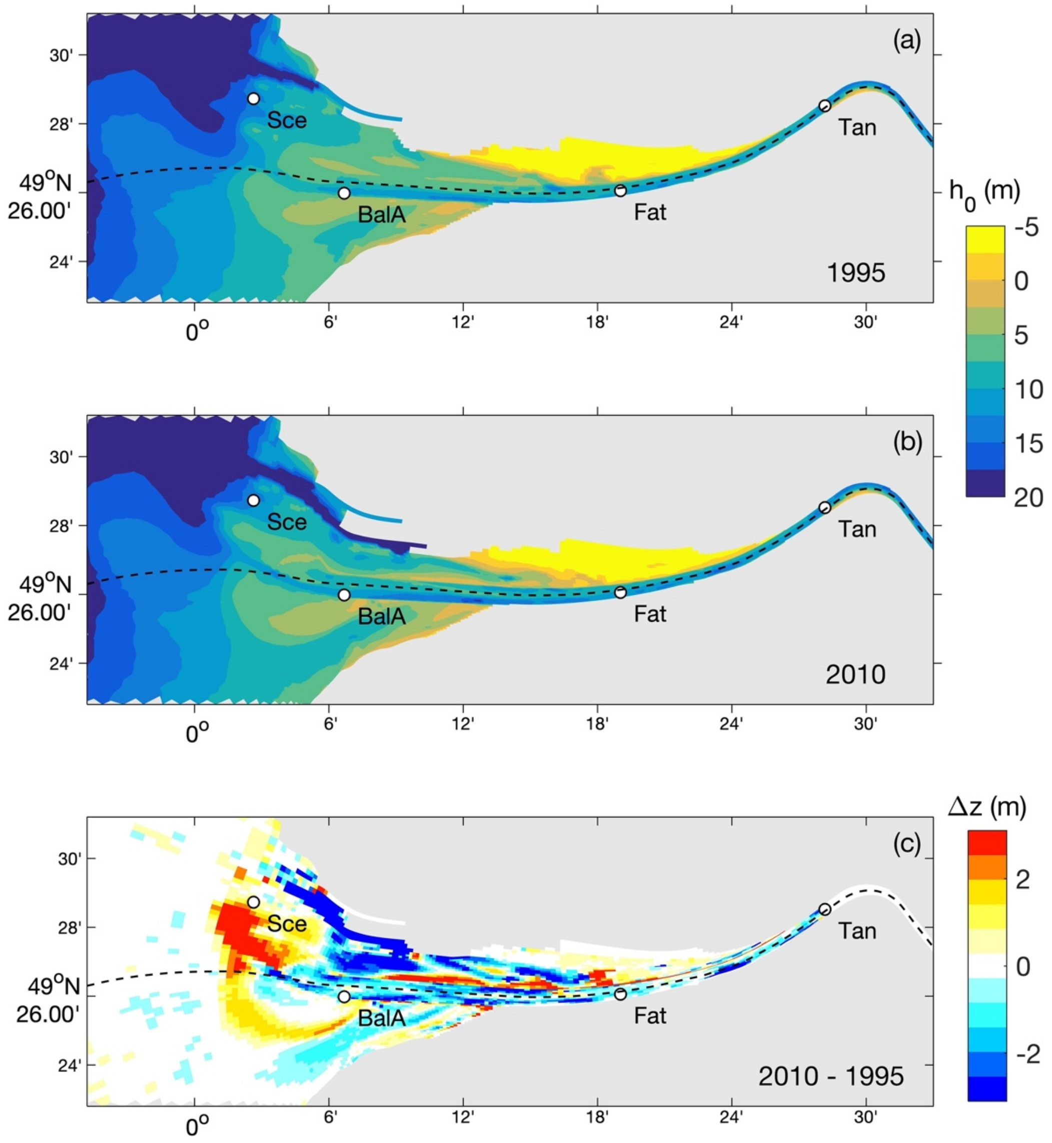

Figure S2. Focuses on the bathymetries of the Seine Estuary mouth (with h_0 the water depth relative to mean sea level): (a) in 1995 and (b) in 2010. (c) Bed level changes Δz (i.e., 2010 – 1995) with erosion for negative values and accretion for positive values. The red line represents a longitudinal transect where water column parameters were computed (Figures S3-S5). The white dots represent Scenes, Balise A, Fatouville, and Tancarville locations ('Sce', 'BalA', 'Fat', and 'Tan', respectively).

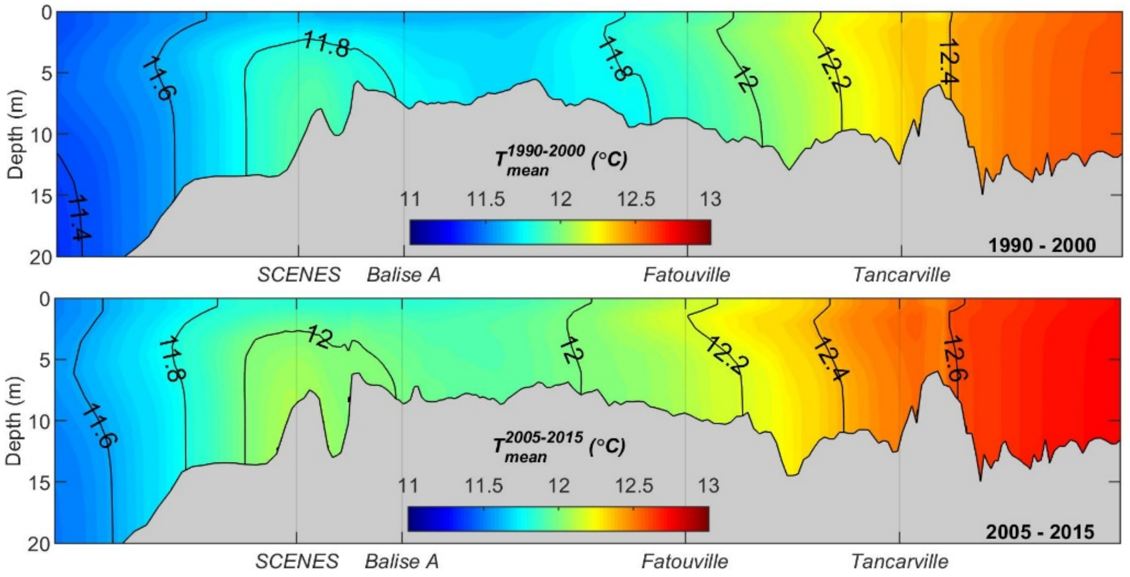

Figure S3. Mean vertical profiles of temperature *T* along the estuary (red transect in Figure S2), averaged over (top panel) the 1990-2000 period and (bottom panel) the 2005-2015 period.

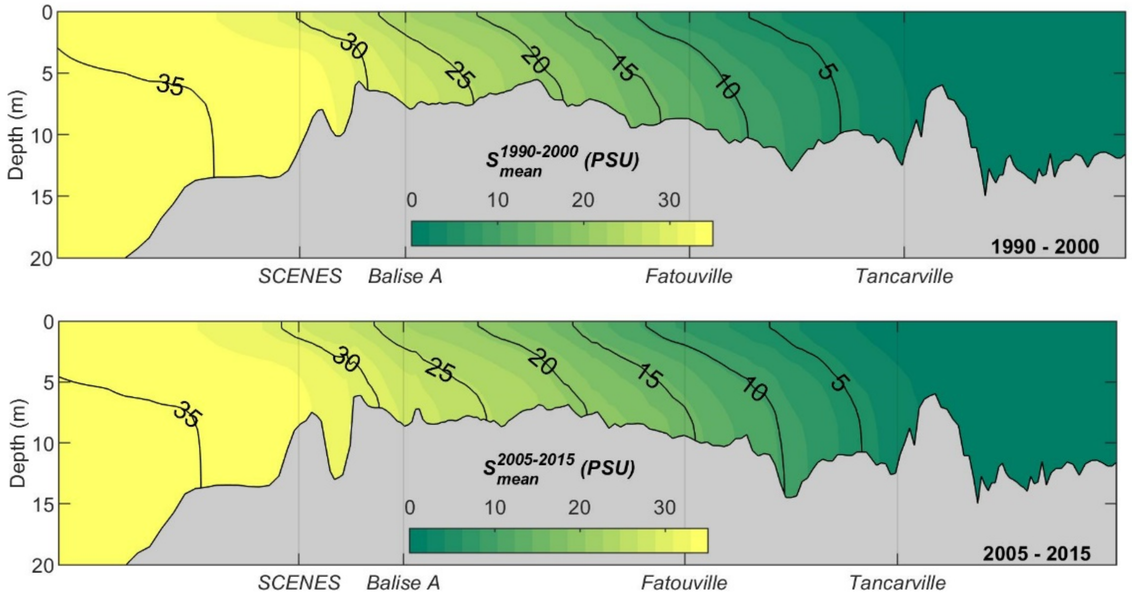

Figure S4. Mean vertical profiles of salinity *S* along the estuary (red transect in Figure S2), averaged over (top panel) the 1990-2000 period and (bottom panel) the 2005-2015 period.

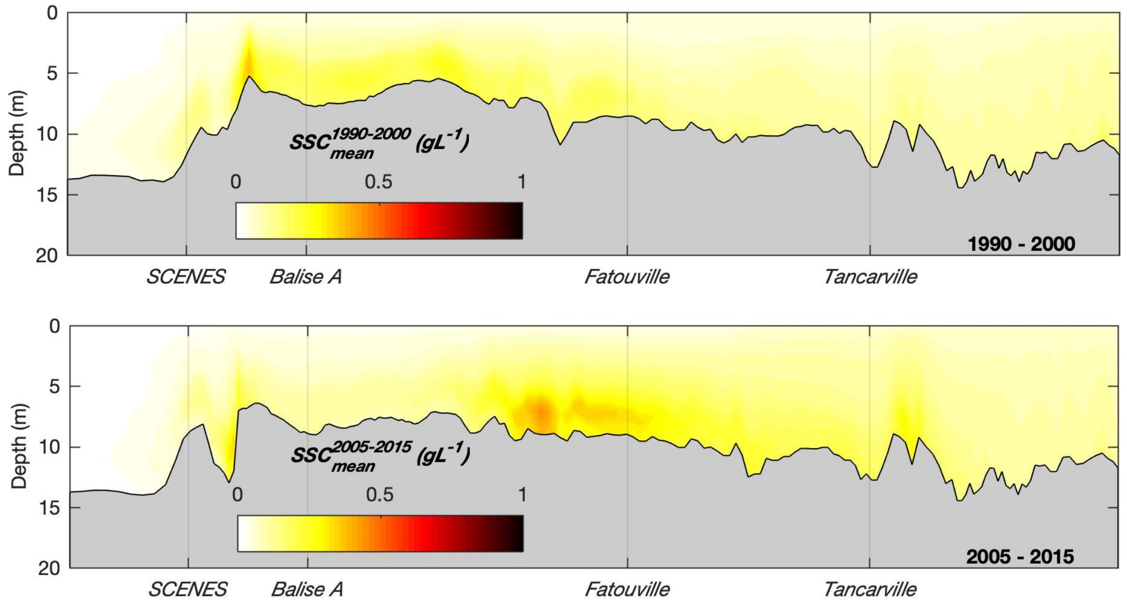

Figure S5. Mean vertical profiles of suspended sediment concentration *SSC* along the estuary (red transect in Figure S2), averaged over (top panel) the 1990-2000 period and (bottom panel) the 2005-2015 period.

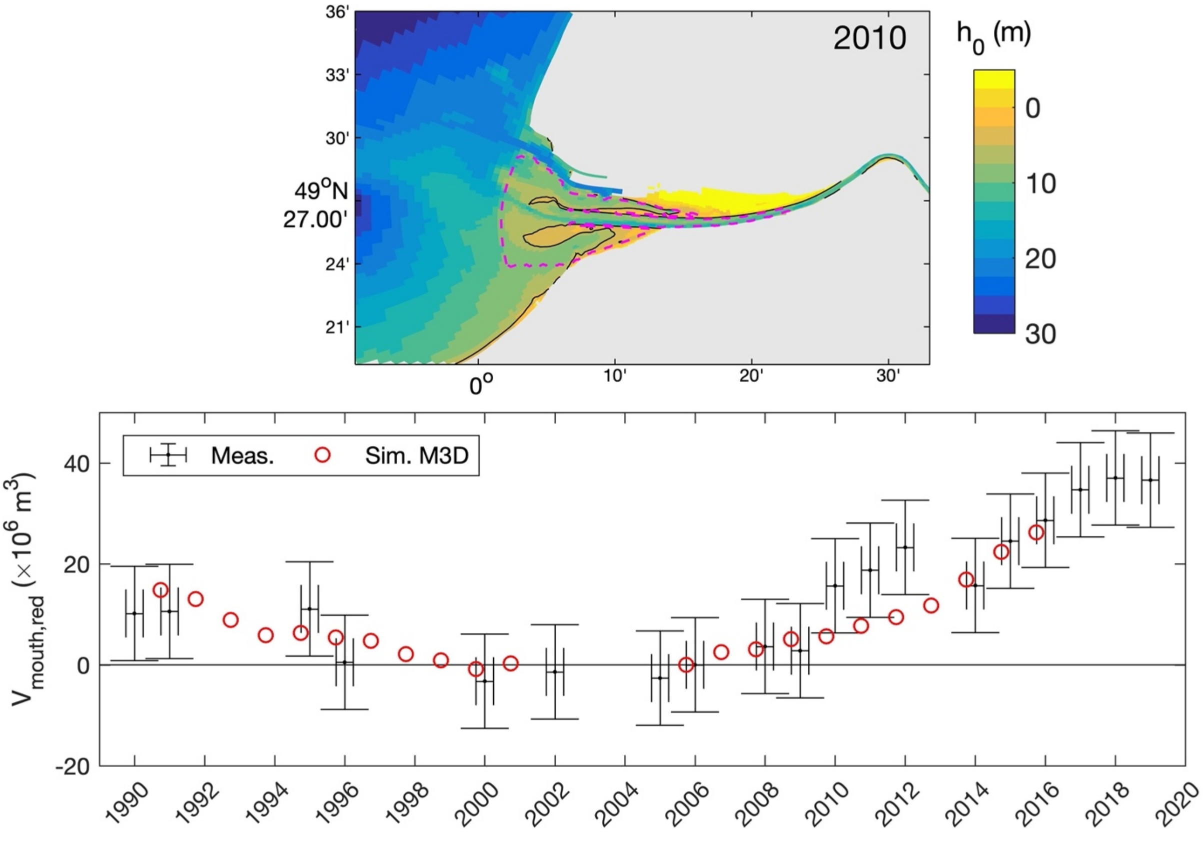

Figure S6. (Top panel) Focus on the bathymetry of the Seine Estuary mouth (with h_0 the water depth relative to mean sea level); the solid black contours represent 5-m isobaths, characterizing intertidal areas. (Bottom panel) Sediment volume $V_{mouth,red}$ in the estuary mouth (magenta dashed contour in top panel), measured from bathymetric surveys (gray brackets), and simulated from the morphostatic model MARS3D 'M3D' used in this study (brown circles).

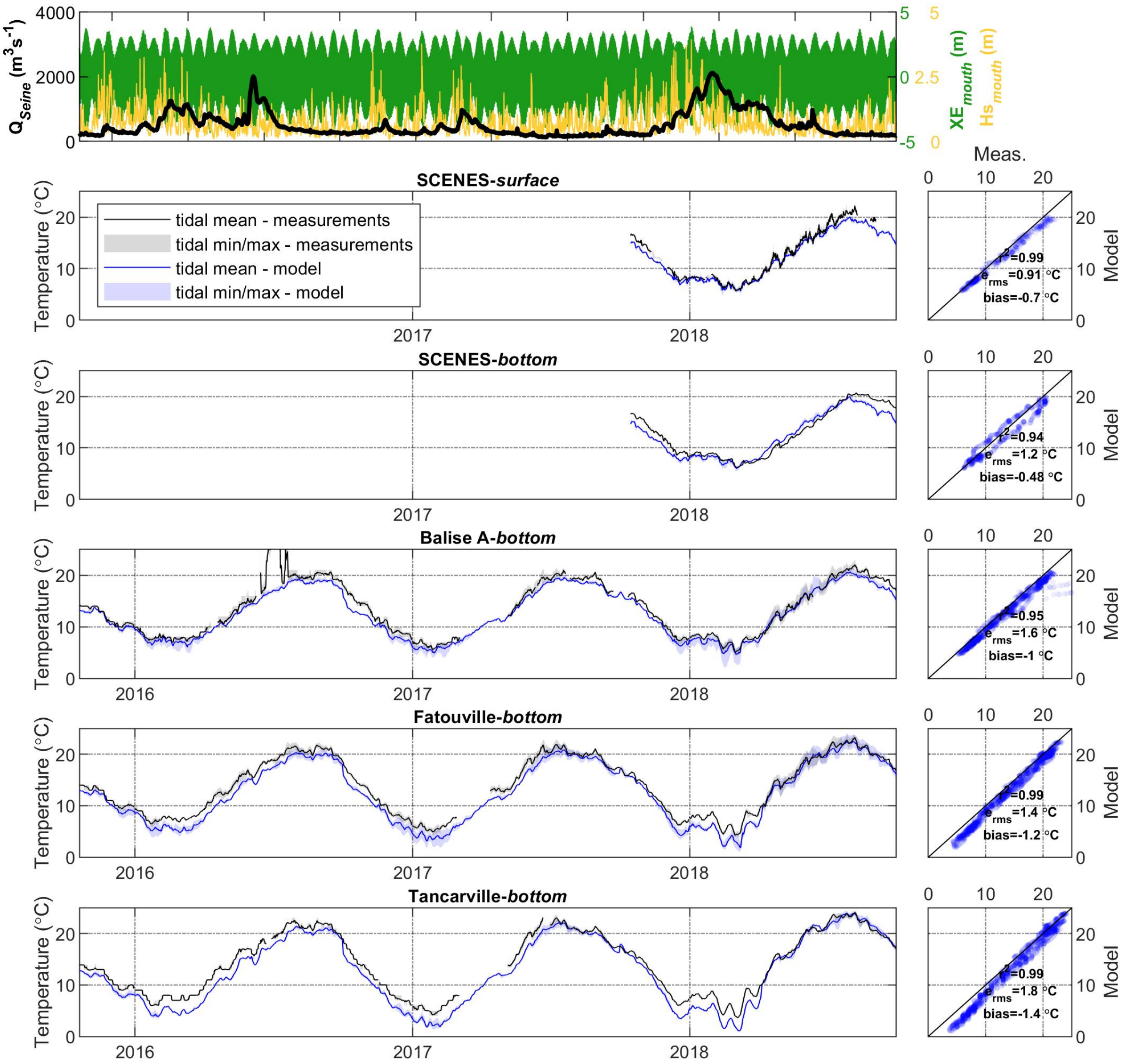

Figure S7. (Top panel) Time series of estuary forcing conditions: Seine River discharge Q_{Seine} (black), significant wave height at the estuary mouth Hs_{mouth} (yellow), and sea surface elevation at the estuary mouth XE_{mouth} (green). Water temperature comparisons between *in situ* measurements (black) from the SYNAPSES monitoring network and simulations (blue) from the MARS3D model used in this study, at four stations along the estuary (Scenes, Balise A, Fatouville, and Tancarville; Figure S2): (left panels) time series and (right panels) correlation plots.

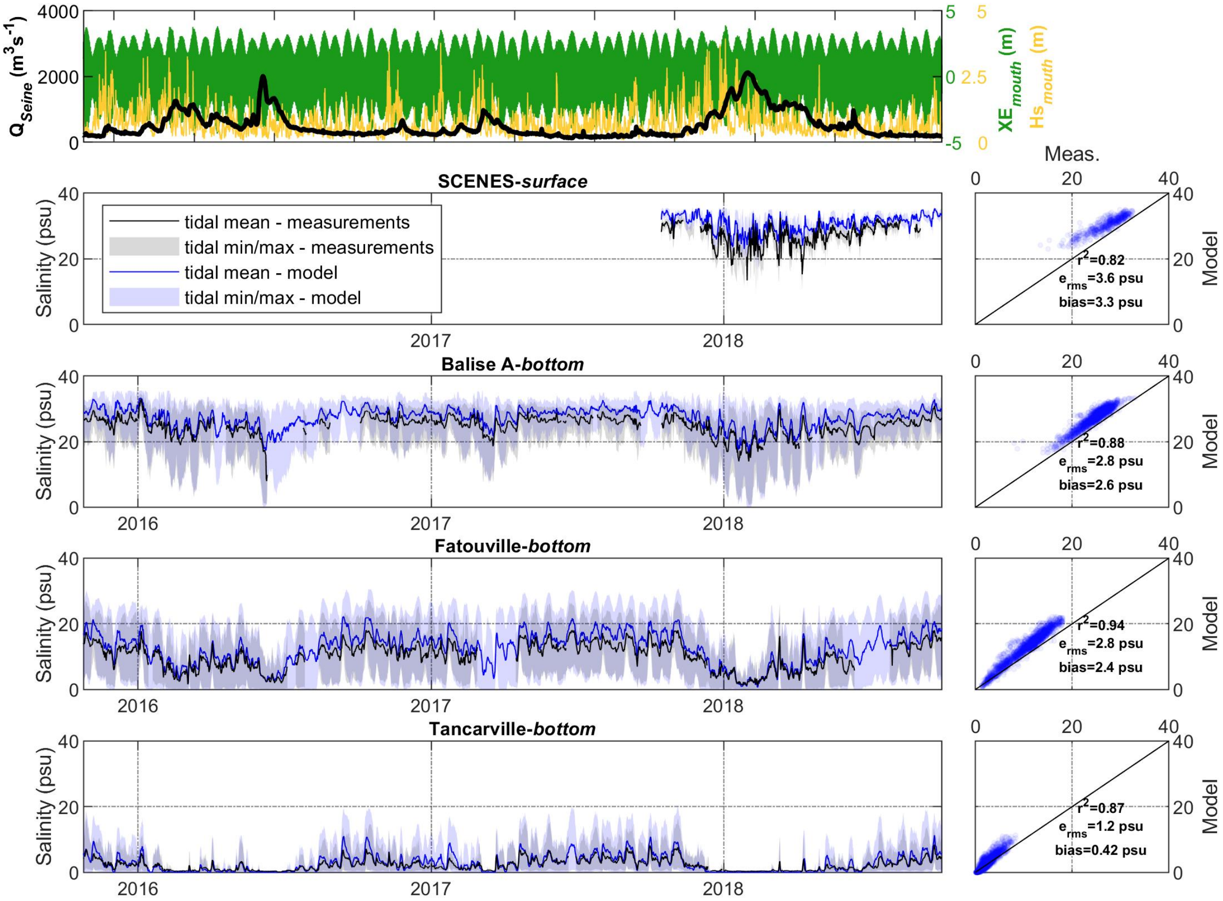

Figure S8. (Top panel) Time series of estuary forcing conditions: Seine River discharge Q_{Seine} (black), significant wave height at the estuary mouth Hs_{mouth} (yellow), and sea surface elevation at the estuary mouth XE_{mouth} (green). Water salinity comparisons between *in situ* measurements (black) from the SYNAPSES monitoring network and simulations (blue) from the MARS3D model used in this study, at four stations along the estuary (Scenes, Balise A, Fatouville, and Tancarville; Figure S2): (left panels) time series and (right panels) correlation plots.

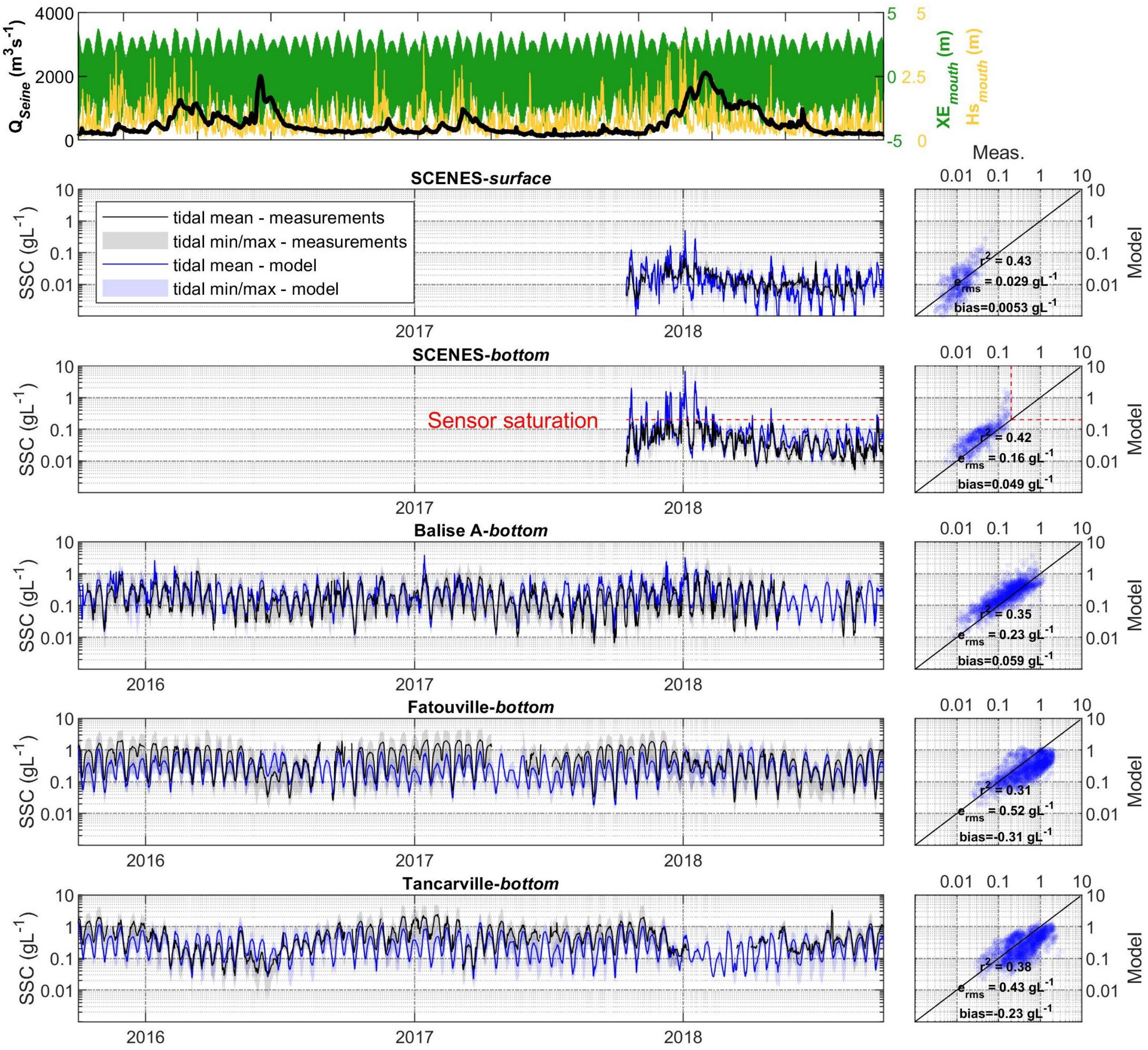

Figure S9. (Top panel) Time series of estuary forcing conditions: Seine River discharge Q_{Seine} (black), significant wave height at the estuary mouth Hs_{mouth} (yellow), and sea surface elevation at the estuary mouth XE_{mouth} (green). Suspended sediment concentration *SSC* comparisons between *in situ* measurements (black) from the SYNAPSES monitoring network and simulations (blue) from the MARS3D model used in this study, at four stations along the estuary (Scenes, Balise A, Fatouville, and Tancarville; Figure S2): (left panels) time series and (right panels) correlation plots.
